# Surgical Treatment of Hydrocephalus

**Published:** 1891-01-31

**Authors:** 


### SURGERY.

### SURGICAL TREATMENT OF HYDROCEPHALUS.

So brilliant have been the results achieved in the new field of brain surgery by Horsley, McEwan, Bergmann, and others, and spina bifida having, as recent cases reported in these columns prove, been brought within the congenital deformities amenable to operation, it is hard to believe that the operative treatment of hydrocephalus, being beset by no technical difficulties, will not some day take its place as a recognised procedure, unsatisfactory though the experience of the past may have been. The symptoms, dullness of intellect gradually increasing until it passes into idiocy or imbecility, blindness, impairment of co-ordination of muscular acts as walking, pareses and contractures, tonic or clonic spasms, &c., are all distinctly due to the presence of the fluid in the ventricles in constantly increasing quantity, raising the intra-cranial pressure, causing wasting of the cerebral substance, and retarding the circulation within the cranium. Could the actual pressure be removed by evacuation of the fluid, and its renewal be prevented, the ossification of the cranium being completed, a cure would be effected. There can be no doubt as to the occasional occurrence of spontaneous cure in slighter cases complicating rickets, the secretion of the fluid subsiding under the influence of the same hygienic and therapeutic measures that serve to arrest the pathological process in the skeleton; the fontanelles gradually close, and mental and bodily health is restored, though the intellectual powers are, so far as our own observations go, either below the average or, if normal, display an irritability or "hyperæsthetic" character. So long as the exudation continues to be poured out, external pressure by bandages can have no effect but to increase the internal pressure on the substance and on the vessels of the brain, with the supervention sooner or later of fatal effects. These remarks are suggested by a case reported by Dr. Pott in the *Jahrb. f. Kinderkrank.*, in which each withdrawal of the fluid was followed by the passing away of the comatose condition, the pupils again reacting to light, the pulse becoming fuller and stronger, the breathing regular, and the desire for food returning. All went on well until pus made its appearance in the cerebro-spinal fluid, and meningitis supervened. Dr. Pott thinks that could suppuration be avoided, incision and drainage would promise more favourable results than any other form of treatment that has been proposed. This seems probable, but all depends on the choice of suitable cases for operation. In congenital hydrocephalus, especially if co-existing with spina bifida, success would appear hopeless, and in extreme forms, in which the brain is already atrophied, it would, even if achieved, result in the saving of an idiot life. The most promising cases, no doubt, are those in which the exudation is chronic and passive rather than acute and active, and an accompaniment of general rickets. In one such case we employed evacuation through the greater fontanelle by a small trocar, sealing the puncture with collodion, and maintaining pressure by bandages. An entire change of diet, from farinaceous "foods" to milk and cream, with cod-liver oil and iodides of iron and potassium, was followed by complete recovery, though for a number of years the child was intensely susceptible to mental excitement and fatigue, and her early education called for the most watchful care.

